# Cardiometabolic index as a predictor of sarcopenia in U.S. adults aged 20–59: results from a cross-sectional study

**DOI:** 10.1016/j.clinsp.2026.100912

**Published:** 2026-03-31

**Authors:** Ji Li, Shou-Jun Bai

**Affiliations:** Department of Nephrology, Qingpu Branch of Zhongshan Hospital Affiliated to Fudan University, Shanghai, China

**Keywords:** Sarcopenia, CMI index, Cross-sectional study, Lipid levels, NHANES

## Abstract

**Background:**

Sarcopenia, characterized by progressive loss of skeletal muscle mass and strength with pathophysiological changes that may originate in early life, and the Cardiometabolic Index (CMI) ‒ a reliable marker for lipid metabolic dysfunction and central adiposity ‒ have associations that are insufficiently studied. This study aimed to investigate the association between CMI and sarcopenia in U.S. adults aged 20–59 and to evaluate the predictive value of CMI for sarcopenia.

**Methods:**

Utilizing data from the National Health and Nutrition Examination Survey (NHANES) spanning 2011–2018, which initially encompassed 39,156 participants, the authors applied age-based screening and exclusion of cases with missing data, resulting in a final analytical cohort of 4784 participants for statistical analysis. Sarcopenia was defined per the Foundation for the National Institutes of Health (FNIH) criteria: Appendicular Lean Mass (ALM) adjusted for BMI < 0.512 in men and < 0.461 in women. CMI was calculated as [triglycerides (mg/dL) / high-density lipoprotein cholesterol (mg/dL)] × [waist circumference (cm) / height (m)] / body mass index (kg/m^2^). Multivariate logistic regression, subgroup analysis (sex, age, race and clinical conditions), Generalized Additive Models (GAM), piecewise regression, Propensity Score Matching (PSM), Inverse Probability Weighting (IPW), and Receiver Operating Characteristic (ROC) curves were employed. Analyses were adjusted for three categories of confounders: sociodemographic factors, behavioral factors, and clinical factors.

**Results:**

Among the 4784 participants, 425 (8.88%) had sarcopenia. Descriptive analysis showed that the median CMI of sarcopenia participants was higher (0.82 vs. 0.43; *p* < 0.001) compared with non-sarcopenia participants, and they had unfavorable demographic/clinical characteristics (e.g., older age, higher prevalence of diabetes). In the full sample, after adjusting for all confounding factors, Elevated CMI correlated with an increased odds of presenting sarcopenia (Odds Ratio [OR = 1.39], 95% CI = 1.05–1.91, *p* = 0.005). The multi-dimensional model incorporating CMI demonstrated superior predictive ability (AUC = 0.819). GAM revealed a non-linear association with a threshold of 0.899, which was strongest in males, individuals aged 20–39 years, non-Hispanic Blacks, those with hypertension, and non-obese or non-diabetic individuals.

**Conclusion:**

Cardiometabolic Index (CMI) has a non-linear positive association with sarcopenia in U.S. young and middle-aged adults, with a threshold of 0.899. The multi-dimensional predictive model incorporating CMI demonstrates superior performance in assessing odds of presenting sarcopenia compared to traditional anthropometric indicators, indicating its potential as a novel clinical biomarker for such evaluations.

## Highlights

• Evaluated the association between the Cardiometabolic Index (CMI) and sarcopenia prevalence among U.S. adults using NHANES data (2011‒2018).
• Found significantly higher CMI values in sarcopenia patients compared to non-sarcopenia patients (0.82 vs. 0.43, *p* < 0.0001).
• Identified a significant positive correlation between increased CMI and sarcopenia prevalence (adjusted OR = 1.39, 95 % CI = 1.05–1.91, *p* = 0.005).
• Confirmed a nonlinear positive correlation between CMI and sarcopenia, with an optimal threshold effect at CMI = 0.899.
• Highlighted the significance of this association in males, younger adults (20‒39 years), non-Hispanic Black individuals, hypertensive, non-obese, and non-diabetic individuals.
• Emphasized the importance of considering CMI as a potential risk factor for sarcopenia and the need for targeted interventions.

## Introduction

The definition of sarcopenia has evolved from initially describing a reduction in muscle mass to being recognized as a distinct musculoskeletal disease, often accompanied by multiple comorbidities.^1^ Two global reviews targeting adults aged 18 and above^2^ and community-dwelling individuals aged 60 and above^3^ reported a pooled sarcopenia prevalence ranging from 5 % to 22 %. This range aligns with findings from a 2016 systematic review, which reported a prevalence of 10 %–40 % based on data from 58 distinct study populations across 26 countries.^4^ Even with the most conservative estimates, the overall prevalence of sarcopenia in the general population remains between 5 % and 10 %.

Sarcopenia can be categorized into primary and secondary types. Primary sarcopenia is primarily associated with age-related physiological changes, whereas secondary sarcopenia often coexists with comorbidities such as diabetes, obesity, and cancer.^5^ This syndrome is significantly linked to various adverse health outcomes, including increased risk of falls, frailty, functional decline, elevated healthcare costs, and higher mortality, exerting profound implications for clinical prognosis^6^ Therefore, identifying and assessing risk factors associated with sarcopenia is crucial for improving patient health and quality of life.

Recent studies have further elucidated the link between obesity and sarcopenia, suggesting that excess adiposity may increase the risk of developing sarcopenia^7^ Although Body Mass Index (BMI) is widely used as an indicator for obesity assessment, its clinical utility is limited by the “obesity paradox”. Accumulating evidence indicates that fat distribution patterns may have a greater impact on cardiovascular disease risk than overall adiposity levels.^8^ Moreover, a close association exists between lipid metabolism and skeletal muscle health. Cross-sectional studies have shown that an elevated Triglyceride to High-Density Lipoprotein Cholesterol (TG/HDL-C) ratio is significantly correlated with reduced relative grip strength in older adults.^9^ Additional research indicates that in middle-aged and older U.S. adults, a higher Non-High-density lipoprotein cholesterol to High-density lipoprotein cholesterol Ratio (NHHR) is associated with an increased likelihood of reduced muscle mass.^10^

Growing evidence supports the role of central obesity and dyslipidemia as key factors in the onset and progression of sarcopenia. Excessive visceral adipose tissue secretes pro-inflammatory cytokines (e.g., tumor necrosis factor-α, interleukin-6), thereby promoting muscle protein breakdown. Concurrently, dyslipidemia impairs mitochondrial function and insulin sensitivity in muscle cells, further exacerbating muscle loss.^11^^,^^12^ The Cardiometabolic Index (CMI), first proposed by Ichiro Wakabayashi et al. in 2015, integrates triglycerides, HDL-C, waist circumference, and BMI to comprehensively reflect central obesity and lipid metabolic status.^7^ Compared to single indicators such as BMI or waist circumference, CMI better captures the synergistic effects between obesity and lipid dysfunction and is regarded as a potentially significant marker for metabolic disorder-related diseases.^13^^,^^14^

Traditionally, sarcopenia has been considered a geriatric syndrome primarily affecting older adults. However, recent evidence suggests that its pathological processes may begin early in life, with deviations from typical age-related changes observable even in younger populations.^1^ Studies also indicate that healthcare costs associated with sarcopenia-related hospitalizations in individuals under 65 may be comparable to or even exceed those in older patients.^15^ Current research on sarcopenia and metabolic biomarkers predominantly focuses on older adults (≥60-years), while studies on young and middle-aged populations (20–59 years) remain scarce. Nonetheless, the presence of sarcopenia in this age group may be linked to long-term adverse outcomes, such as disability in later life, and presents opportunities for intervention. Thus, identifying early predictive markers like CMI could facilitate targeted preventive strategies.

Based on this background, the present study aims to investigate the association between CMI and sarcopenia in U.S. adults aged 20–59 years. Utilizing data from the National Health and Nutrition Examination Survey (NHANES) 2011–2018, the authors will assess the relationship between CMI and the prevalence of sarcopenia. To our knowledge, this is the first study to systematically examine the association between CMI and increased odds of presenting sarcopenia in this age group.

## Methods

### Study population

**Fig. 1:**
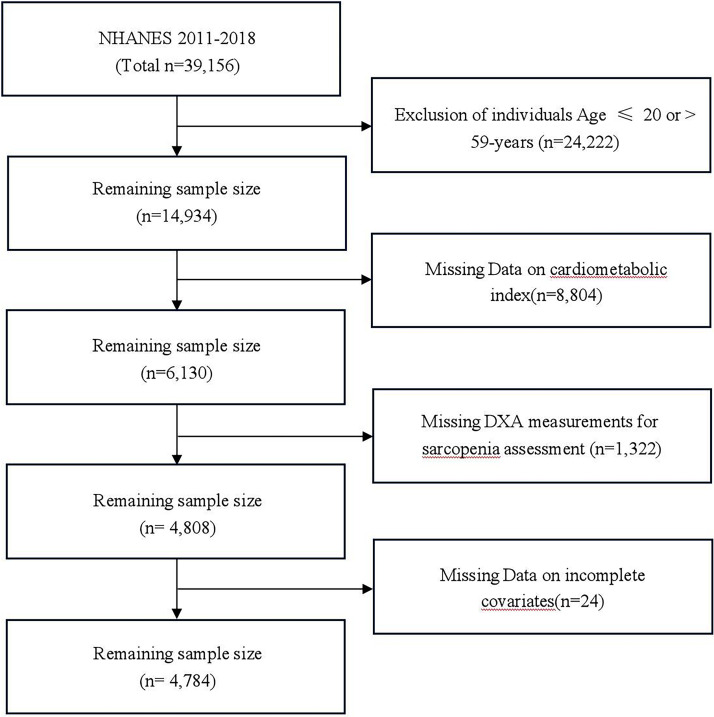
Flow chart of participant select.

NHANES is a nationally representative survey of U.S. residents using multistage probability sampling. The authors included 2011–2018 NHANES data (initial n = 39,156). Exclusions were: 1) Age ≤ 20 or ≥ 59-years (n = 24,222); 2) Missing CMI components (n = 8,804); 3) Missing DXA measurements for sarcopenia assessment (n = 1,322); 4) Missing covariate data (e.g., education, smoking, chronic diseases; n = 24). The final sample included 4784 participants (425 with sarcopenia). The participant selection flow chart is shown in Fig. 1.

### Exposure and outcome definitions

CMI (Exposure): Calculated as . Treated as a continuous variable at baseline.

Sarcopenia (Outcome): Assessed via dual-energy X-Ray Absorptiometry (DXA) (excluding pregnant participants, weight > 136 kg, or height > 196 cm). Appendicular Lean Mass (ALM) was defined as the combined upper/lower limb muscle mass. Sarcopenia was diagnosed using the Foundation for the National Institutes of Health (FNIH) criteria: Sarcopenia index (SAR) = ALM/BMI < 0.789 (men) and < 0.512 (women) .^16^

### Study variables

Covariate selection was based on prior literature and clinical relevance, encompassing sociodemographic, behavioral, and clinical factors (for details, see Supplementary Table 3). Sociodemographic: Age, sex (male/female), race/ethnicity (Mexican American, Other Hispanic, Non-Hispanic White, Non-Hispanic Black, Other Race), education (Under high school, completed high school, College or above), marital status (Married, Widowed, Never married), poverty income ratio (PIR: ≤ 1.3, 1.3–3.5, > 3.5). Behavioral: Smoking (Yes/No), alcohol consumption (Yes/No), physical activity (PA): Assessed via NHANES questionnaire, the total amount of PA was assessed by summing minutes of activity per week multiplied by the Metabolic Equivalent of Task (MET) score of each activity: low (0 to < 600 Met-min/week), medium (600 to < 1200 Met-min/week), and high (≥ 1200 Met-min/week). Clinical: Hypertension (Yes/No), diabetes (Yes/No/Unclear), Coronary Artery Disease (CAD; Yes/No), cancer (Yes/No), asthma (Yes/No), BMI categories (< 25, 25–29.9, ≥ 30 kg/m^2^). Biochemical/Dietary: TG, HDL-C, Total Cholesterol (TC); Dietary intake data were collected using the 24-hour dietary recall method from NHANES. All participants completed two interviews: the first being a face-to-face interview conducted at mobile examination centers, and the second a telephone follow-up conducted 3 to 10 days thereafter. The processing and definitions of nutritional intake variables ‒ including energy, protein, carbohydrates, and fats ‒ strictly adhered to the NHANES official survey methodology and analytical guidelines (https://www-n.cdc.gov/nchs/nhanes/AnalyticGuidelines).

### Statistical analysis

Analyses were performed using R 4.2.0, accounting for NHANES’ complex sampling design with modified weights (original 2-year weights/4). Descriptive Statistics: Continuous variables (normal distribution: weighted mean ± 95 % Confidence Intervals (95 % CI); non-normal: median ± Interquartile Ranges [IQR]); categorical variables: weighted proportions ± 95 % CI. Group differences were tested via weighted chi-square (categorical), ANOVA (normal continuous), or Kruskal-Wallis H (skewed continuous). Multivariable logistic regression analysis was performed to assess the association between CMI and the odds of sarcopenia. Three models: a) Unadjusted; b) Adjusted for sociodemographic factors (age, sex, race, education, marital status); c) Fully adjusted (Model 2 + clinical/behavioral/dietary/biochemical covariates). Non-linear and Threshold Analyses: Generalized Additive Models (GAM) with logistic link function to test non-linearity. Piecewise regression (recursive algorithm minimizing AIC) to identify thresholds, validated via likelihood ratio test. Predictive Performance: Receiver Operating Characteristic (ROC) curves to compare CMI, BMI, and WC. Optimal cut-offs determined by Youden’s index. Sensitivity Analyses: Propensity Score Matching (PSM) (1:1 nearest neighbor, caliper = 0.01, adjusting for all covariates), Inverse Probability Weighting (IPW), CMI categorization (quartiles), and multiple imputation for missing data. Subgroup Analyses: Stratified by sex, age, race, BMI, diabetes, hypertension, and PA. Interaction effects tested via log-likelihood ratio tests. Statistical significance was set at *p* < 0.05.

## Results

### Characteristics of the study population

This study ultimately enrolled 4784 young and middle-aged participants, of whom 425 were diagnosed with sarcopenia, yielding an overall prevalence rate of 8.88 % (refer to Fig. 1 for the detailed participant screening process). The sample comprised 50.86 % males and 49.14 % females, indicating a generally balanced sex distribution. Weighted baseline characteristics stratified by sarcopenia status are summarized in Table 1. Regarding demographic characteristics, the sarcopenia group exhibited a higher mean age, with a predominant racial composition of non-Hispanic Whites and Mexican Americans. Additionally, this group demonstrated overall lower socioeconomic status and educational attainment. In terms of lifestyle factors, the sarcopenia group displayed lower levels of physical activity. Nutritional intake analysis revealed that protein, fat, and total energy consumption were generally reduced in this group. Anthropometric data indicated that both BMI and WC were elevated in the sarcopenia group compared to the non-sarcopenia group. With respect to biochemical markers, the sarcopenia group showed higher levels of TC and TG, along with lower HDL-C levels. Furthermore, the prevalence rates of diabetes and hypertension were significantly higher in the sarcopenia group. Regarding the CMI, participants with sarcopenia exhibited a significantly higher median CMI value than those without sarcopenia (0.82 vs. 0.43; *p* < 0.001).

**Table 1:** Baselines characteristics of participants, weighted.

**Characteristic**	**Non-sarcopenia**	**Sarcopenia**	**P**
Gender ( %)	4359	425	0.2346
Male	50.46	54.91	
Female	49.54	45.09	
Age (median [range]) (years)	39.00 [20.00, 59.00]	45.00 [20.00, 59.00]	<0.0001
Race ( %)			<0.0001
Mexican American	9.43	24.88	
Other Hispanic	7.06	13.17	
Non-Hispanic White	62.69	49.68	
Non-Hispanic Black	11.24	2.87	
Other Race	9.58	9.40	
education ( %)			<0.0001
Under high school	3.42	14.36	
Completed high school	9.37	14.07	
College or above	87.21	71.56	
Marital status ( %)			0.5508
Married	51.81	53.66	
Widowed	1.27	0.83	
Never married	46.92	45.51	
alcohol ( %)			0.0076
Yes	84.68	75.44	
No	15.32	24.56	
Hypertension ( %)			0.0001
Yes	22.52	34.63	
No	77.48	65.37	
Diabetes ( %)			<0.0001
Yes	5.56	13.43	
No	92.89	82.09	
Unclear	1.55	4.49	
smoke ( %)			0.856
Yes	41.96	42.59	
No	58.04	57.41	
CAD ( %)			0.4916
Yes	0.99	1.46	
No	99.01	98.54	
cancer ( %)			0.8463
Yes	4.52	4.79	
No	95.48	95.21	
asthma ( %)			
Yes	14.74	17.44	0.2037
No	85.26	82.56	
CMI (median [range])	0.43 [0.03, 31.09]	0.82 [0.08, 16.08]	<0.0001
PA ( %)			0.0497
Vigorous	17.56	13.25	
Moderate	12.75	10.56	
Mild	69.69	76.18	
PIR ( %)			<0.0001
<1.3	23.08	36.73	
≥1.3 / <3.5	35.29	37.42	
≥3.5	41.64	25.85	
Total Protein (median [range]) (g)	80.28 [2.12, 454.48]	70.65 [20.44, 233.33]	0.0013
Total Carbohydrate (median [range]) (g)	242.68 [6.14, 1122.62]	225.06 [55.44, 1052.56]	0.0689
Total Dietary fiber(median[range]) (g)	15.70 [0.15, 95.20]	14.96 [1.80, 65.45]	0.1007
Total fat (median [range]) (g)	78.05 [0.75, 418.26]	69.82 [7.15, 200.82]	0.0046
Total Energy (median [range]) (kcal)	2071.00 [96.50, 9479.50]	1880.31 [440.00, 5564.00]	0.0019
Total sugar (median [range]) (g)	96.66 [0.13, 680.34]	92.92 [4.92, 892.64]	0.2672
Serum creatinine (median[range]) (µmoL/)	75.15(20.64)	69.68(17.59)	0.0099
TC (median [range]) (mmoL/L)	4.81 [2.07, 15.83]	5.04 [2.40, 10.06]	0.0002
TG (median [range]) (mmoL/L)	1.03 [0.11, 47.79]	1.42 [0.27, 15.88]	<0.0001
HDL (median [range]) (mmoL/L)	1.32 [0.26, 4.47]	1.16 [0.16, 2.64]	<0.0001
WC (median [range]) (cm)	95.00 [63.10, 160.80]	108.40 [67.20, 176.00]	<0.0001
BMI (median [range]) (kg/m^2^)	27.30 [15.50, 60.90]	33.90 [17.80, 61.70]	<0.0001
SAR (median [range])	0.83 [0.51, 1.47]	0.66 [0.38, 0.79]	<0.0001

For continuous variables: survey-weighted mean (95 % CI), p-value was by survey-weighted logistic regression (svyglm).

For categorical variables: survey-weighted percentage (95 % CI), p-value was by survey-wei-ghted Chi-Square test (svytable).

CAD, Coronary Artery Disease; CMI, Cardiometabolic Index; PIR, Ratio of family Income to Poverty; PA, Physical Activity; TC, Total Cholesterol; TG, Triglycerides; HDL-C, High-Density Lipoprotein Cholesterol; WC, Waist Circumference; BMI, Body Mass Index; SAR, Sarcopenia Index.

### Association between CMI and sarcopenia

**Fig. 2:**
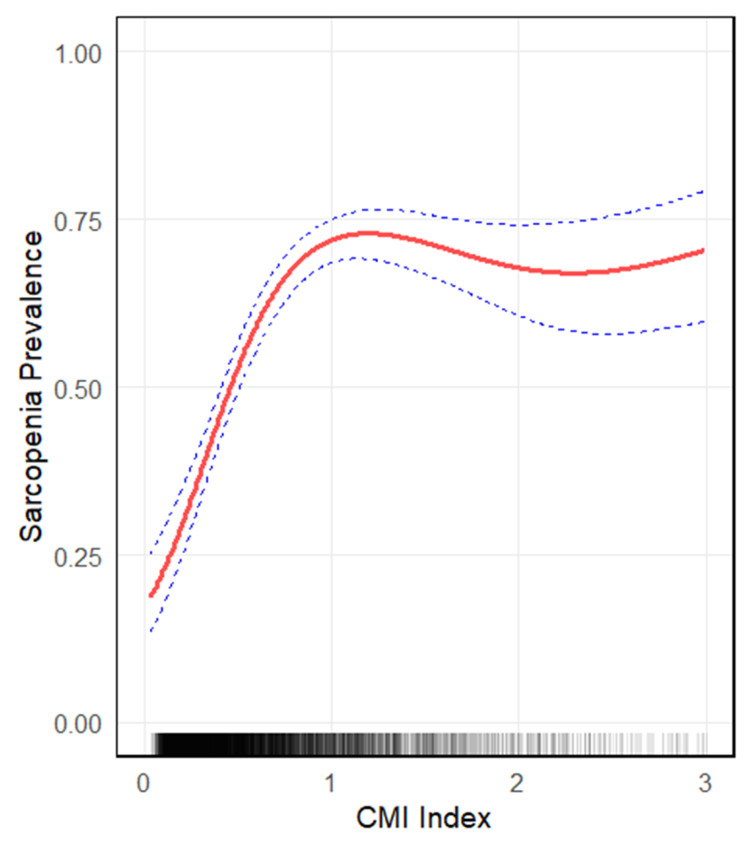
Generalized Additive Model (GAM) combined with smooth curve fitting showing the non-linear association between CMI and the odds of sarcopenia (adjusted for covariates [Refer to Supplementary Table 3]). Solid line: predicted odds; Dotted lines: 95 % CI; Grayscale strip: CMI distribution density (darker = higher density). CMI, Cardiometabolic Index.

Multivariate logistic regression showed a positive CMI-sarcopenia association (Table 2): Model 1 (Unadjusted): OR = 1.29 (95 % CI: 1.19–1.39; *p* < 0.0001); Model 2 (Sociodemographic-adjusted): OR = 1.21 (95 % CI: 1.12–1.31; *p* = 0.0004); Model 3 (Fully adjusted): OR = 1.39 (95 % CI: 1.05–1.91; *p* = 0.005). GAM revealed a non-linear association (Fig. 2). A threshold of 0.899 was identified (Table 3): CMI < 0.899 had a stronger association (OR = 1.18, 95 % CI: 1.14–1.22) than CMI ≥ 0.899 (OR = 1.01, 95 % CI: 0.99–1.02; logarithmic Likelihood Ratio Test (LRT) *p* < 0.0001).

**Table 2:** Logistic regression analysis between CMI index with sarcopenia prevalence.

**Characteristic**	**Model 1 OR (95 % CI)**	**Model 2 OR (95 % CI)**	**Model 3 OR (95 % CI)**	**Model 4 OR (95 % CI)**
CMI	1.29 (1.19, 1.39)	1.21 (1.12, 1.31)	1.39 (1.05, 1.91)	^△^1.44 (0.92, 2.25)
Categories				
Lower	1	1	1	1
Higher	1.28 (1.16, 1.41)	1.21 (1.09, 1.33)	1.57 (1.16, 2.13)	★2.82 (2.03, 3.93)
P for trend	<0.0001	0.0004	0.005	<0.0001

Model 1 was adjusted for no covariates;

Model 2 was adjusted for age, gender, race, marital status and education;

Model 3 was adjusted for covariates in Model 2 + diabetes, blood pressure, PIR, total protein, Carbohydrate, Dietary fiber, total kcal, total sugar, total fat, smoked, physical activity, alcohol use, coronary artery disease, asthma, cancers, WC, BMI, HDL, TC and TG were adjusted.

Model 4 ^△^ PSM analysis only in model 4.

CMI, Cardiometabolic Index; PSM, Propensity Score Matching; IPW, Inverse Probability Weighting; BMI, Body Mass Index; WC, Waist Circumference; TC, Total Cholesterol; TG, Triglycerides; HDL-C, High-Density Lipoprotein Cholesterol; OR, Odds Ratio.

★ IPW analysis only in model 4.

**Table 3:** Two-piecewise logistic regression and logarithmic likelihood ratio test explained the threshold effect analysis of CMI index with sarcopenia prevalence.

**CMI**	**ULR Test**	**PLR Test**	**LRT test**
	**OR (95 % CI)**	**OR (95 % CI)**	**p-value**
<0.899	1.03 (1.02, 1.04)	1.18 (1.14, 1.22)	<0.0001
≥0.899		1.01 (0.99, 1.02)	

CMI, Cardiometabolic Index; ULR, Univariate Logistic Regression; PLR, Piecewise Logistic Regression; LRT, Logarithmic Likelihood Ratio Test; OR, Odds Ratio; Statistically significant: *p* < 0.05.

### Sensitivity analysis

To evaluate the consistency of the observed association between CMI and sarcopenia, the study employed multiple statistical approaches for sensitivity analyses. An additional 1:1 PSM analysis indicated that the direction of the association remained consistent but was not statistically significant (OR = 1.44, 95 % CI: 0.92–2.25; see Table 2 and Supplementary Table 1). When CMI was categorized as a binary variable and analyzed using logistic regression, results revealed that individuals in the highest CMI group had a 57 % higher odds of sarcopenia compared to the lowest group, with an OR of 1.57 (95 % CI: 1.16–2.13). A trend p-value of 0.005 suggested a statistically significant dose-response relationship. Furthermore, IPW applied to the binary CMI variable demonstrated a substantially elevated risk of sarcopenia in the highest CMI group ‒ 182 % higher than the lowest group ‒ yielding an OR of 2.82 (95 % CI: 2.03–3.93; see Table 2 and Supplementary Table 2). Collectively, these findings consistently support a positive correlation between CMI and the likelihood of sarcopenia onset.

### ROC curve analysis for predictive modeling

**Fig. 3:**
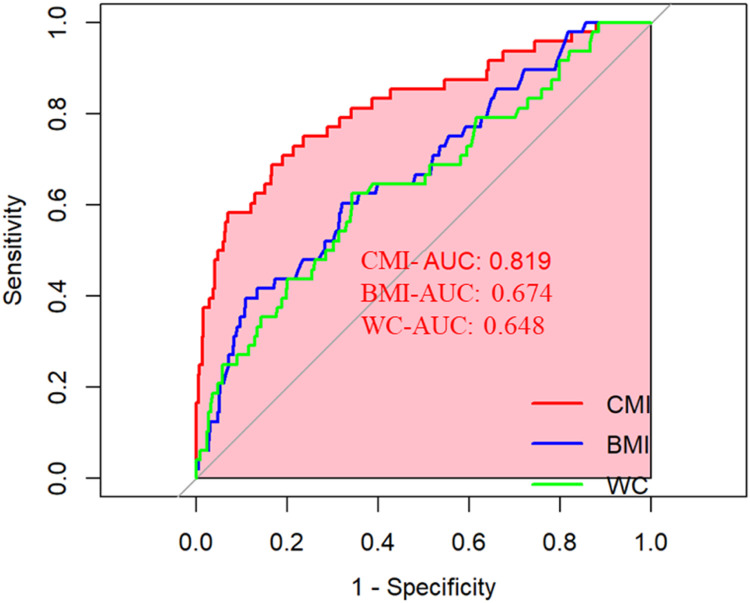
ROC curves for CMI, BMI and WC prediction of sarcopenia. CMI, Cardiometabolic Index; BMI, Body Mass Index; WC, Waist Circumference.

To establish the final predictive model for sarcopenia, this study integrated a broad range of covariates across multiple categories. These encompassed demographic factors (age, gender, race, marital status, education level, PIR, medical conditions (diabetes, hypertension, CAD, asthma, cancer), lifestyle behaviors (smoking history, PA level, alcohol consumption), dietary intake metrics (total energy intake, total sugar, total fat, carbohydrates, dietary fiber), and biochemical parameters (TC, HDL-C, TG). The assessment of the model’s predictive performance, as shown in Fig. 3, revealed a high level of discrimination, with an AUC of 0.819. At the optimal cut-off point of 0.114, the model achieved a sensitivity of 68.75 % and a specificity of 83.33 %. When compared to traditional anthropometric measures ‒ BMI and WC ‒ the proposed model demonstrated enhanced predictive capability. Specifically, the AUC of the CMI-based model (0.819) was significantly greater than that of BMI (0.674) and WC (0.648). In terms of sensitivity, the CMI model (68.75 %) surpassed both BMI (39.58 %) and WC (62.50 %). Regarding specificity, the CMI model (83.33 %) was slightly lower than BMI (89.09 %) but markedly higher than WC (65.67 %). The optimal threshold values for BMI and WC were determined to be 35.95 and 101.35, respectively. These results suggest that the multi-dimensional model incorporating CMI holds strong promise for application in clinical settings, particularly for early identification and risk stratification of sarcopenia.

### Subgroup analysis

To assess the heterogeneity of the association between CMI and sarcopenia across different populations, the authors performed stratified analyses and interaction tests (Table 4). Formal tests for interaction revealed that, with the exception of BMI (p for interaction = 0.009), no significant modifying effects on the CMI-sarcopenia association were observed for gender, age, race, physical activity level, or history of hypertension or diabetes (all p for interaction > 0.05). Based on the significant interaction effect identified for BMI, the authors further conducted stratified analyses. The results indicated that in the normal-weight group (BMI < 25 kg/m^2^), each one-unit increase in CMI was associated with a 2.28-fold increased risk of sarcopenia (OR = 2.28, 95 % CI: 1.35–3.84, *p* = 0.002). In the obese group (BMI ≥ 30 kg/m^2^), the corresponding risk increase was 1.1-fold (OR = 2.10, 95 % CI: 1.15–3.82, *p* = 0.016). However, no statistically significant association was detected in the overweight group (BMI 25–29.9 kg/m^2^). Although interaction tests for the remaining covariates did not reach statistical significance, the authors report trend differences observed in other subgroups for completeness. For instance, the strength of the association appeared stronger in males (OR = 2.27) than in females, higher in non-diabetic individuals (OR = 2.40) compared to those with diabetes, and slightly greater in the 20–39 age group (OR = 2.31) relative to the 40–59 age group (OR = 2.24). These findings may offer preliminary insights into potential high-risk subgroups; however, their precise modifying roles require confirmation in larger-scale studies.

**Table 4:** Subgroup regression analysis between CMI index with sarcopenia prevalence.

**Variable**	**Percent**	**OR**	**Lower**	**Upper**	**p**	**p for interaction**
Overall	100	1.28	1.16	1.41	<0.001	
Stratified by gender						0.966
female	50.4	1.26	0.75	2.12	0.391	
male	49.6	1.27	1.14	1.42	<0.001	
Stratified by age(years)						0.674
20‒39	49.9	1.31	1.14	1.5	<0.001	
40‒59	50.1	1.24	1.05	1.45	0.012	
Stratified by race						0.667
Mexican American	15	1.2	1.06	1.36	0.009	
Other Hispanic	10.7	1.15	0.88	1.51	0.303	
Non-Hispanic White	35.3	1.28	1.15	1.44	<0.001	
Non-Hispanic Black	20	1.78	1.34	2.37	<0.001	
Other Race	19	1.26	1.09	1.46	0.003	
Stratified by BMI (kg/m^2^)						0.009
< 25	33.1	2.28	1.38	3.75	0.002	
25‒29.9	31.4	1.18	0.99	1.39	0.067	
≥ 30	35.5	1.1	1.02	1.2	0.016	
Stratified by diabetes						0.625
Yes	7.9	1.05	0.93	1.17	0.447	
No	90.4	1.4	1.19	1.63	<0.001	
Unclear	1.7	0.98	0.79	1.23	0.888	
Stratified by hypertension						0.389
Yes	23.9	1.31	1.12	1.53	0.001	
No	76.1	1.2	1.04	1.37	0.013	
Stratified by PA						0.28
Vigorous	17.5	1.48	1.21	1.61	<0.001	
Moderate	12.4	1.12	0.97	1.33	0.122	
Mild	70	1.28	1.15	1.44	<0.001	

BMI, Body Mass Index; PA, Physical Activity; OR, Odds Ratio.

## Discussion

This study examined the relationship between CMI and sarcopenia in U.S. adults aged 20–59 using multiple statistical methods, including multivariate logistic regression, GAM, smooth curve fitting, and sensitivity and subgroup analyses. Among 4784 participants aged 20–59 years, the prevalence of sarcopenia was 8.88 %, consistent with previous studies,^2^^,^^17^ which supports the reliability of the findings. The results also showed that individuals with sarcopenia had higher CMI levels, suggesting a meaningful link between CMI and sarcopenia.

This study presents the first evidence of a statistically significant positive link between CMI and odds of presenting sarcopenia in young and middle-aged adults. The association was consistent across both the unadjusted model (Model 1) and the fully adjusted model (Model 3), which accounted for all potential confounders. In Model 3, each one-unit increase in CMI was associated with a 39 % higher odds of presenting sarcopenia (OR = 1.39, 95 % CI: 1.05–1.91). Further analysis using GAM and smooth curve fitting revealed a non-linear relationship between CMI and odds of presenting sarcopenia. An inflection point was identified at a CMI value of 0.899, where the nature of the association changed significantly: below this threshold, the link was stronger, but weakened above it. The likelihood ratio test confirmed that this threshold effect was highly significant (*p* < 0.0001), supporting its reliability. These findings offer a quantitative basis for considering CMI as a potential clinical screening tool.

This study used 1:1 PSM to further validate the link between CMI and sarcopenia while minimizing confounding. The analysis found that each one-unit increase in CMI was associated with a 44 % higher odds of presenting sarcopenia (OR = 1.44, 95 % CI: 0.92–2.25). Although not statistically significant (*p* > 0.05), this trend aligned with the main findings, suggesting CMI may still serve as a relevant risk factor. The lack of significance could be due to a smaller sample size and residual confounding after matching. When analyzed as a binary variable, individuals in the high CMI group had a significantly greater risk ‒ 57 % higher than those in the low group (OR = 1.57, 95 % CI: 1.16–2.13; p-trend = 0.005). IPW analysis revealed an even stronger association, with a 182 % increased risk in the high CMI group (OR = 2.82, 95 % CI: 2.03–3.93), which was highly significant and supported a more causal interpretation. Across all methods, results consistently showed a positive relationship between CMI and sarcopenia, regardless of whether CMI was modeled continuously or categorically. Notably, the risk was most pronounced among individuals with elevated CMI levels, highlighting its potential as a clinical predictor. Despite wide CI in the PSM analysis, consistent sensitivity results support the reliability of these findings and lay a foundation for developing an odds of presenting sarcopenia prediction model based on CMI. Further prospective studies are needed to confirm this association.

The relationship between obesity-related metrics and sarcopenia continues to be a topic of discussion within the scientific community. Accumulating evidence indicates that higher BMI may have a protective effect against sarcopenia,^18^, ^19^, ^20^ while elevated body fat percentage is generally considered a risk factor for the condition.^19^^,^^21^ This apparent contradiction, known as the “obesity paradox”, could be attributed to age-associated changes in body composition among older individuals, including reduced skeletal muscle mass concurrent with increased adiposity. In this context, body weight and BMI may remain stable, yet there is a significant increase in body fat content and a redistribution of fat, such as the accumulation of visceral fat and the infiltration of muscle tissue by adipose deposits.^22^^,^^23^

Visceral fat accumulation may play a role in promoting sarcopenia through the induction of chronic inflammatory responses.^24^ In parallel, sarcopenia can result in decreased PA levels, which further facilitates fat accumulation, thereby establishing a reinforcing feedback loop.^25^^,^^26^ Consequently, commonly used obesity evaluation indicators ‒ such as BMI and WC ‒ exhibit significant limitations. BMI lacks the ability to distinguish between fat mass and lean body mass, two components that carry different health implications. Although WC reflects visceral fat content to some extent, it fails to consider individual differences in total body weight.

In contrast, the CMI offers a comprehensive evaluation of lipid metabolic disturbances and central adiposity through its calculation formula (TG/HDL-C × WC/height). The multidimensional predictive model constructed based on CMI in this study demonstrated superior predictive capability (AUC = 0.819) compared to both BMI and WC. These results correspond with findings from a Korean study indicating that “women with visceral obesity exhibit a more substantial decline in limb skeletal muscle mass”,^23^ as well as observations from a Japanese cohort demonstrating that “visceral fat accumulation is associated with reduced muscle mass” among individuals with type 2 diabetes mellitus,^27^ thereby reinforcing the clinical utility of CMI in assessing odds of presenting sarcopenia.

The construction of the CMI reflects the synergistic influence of lipid metabolic dysfunction and central obesity. Our results reveal that individuals diagnosed with sarcopenia generally display increased levels of TC and TG, coupled with decreased concentrations of HDL-C. These findings suggest that disturbances in lipid metabolism may contribute to the pathophysiological processes underlying sarcopenia. Earlier studies have confirmed an inverse correlation between appendicular skeletal muscle mass and TG levels, as well as a positive association with HDL-C.^28^ Lee et al. also observed that relative handgrip strength is inversely related to cardiometabolic risk indicators such as elevated TG and reduced HDL-C.^11^ In sarcopenic patients, lipid infiltration within muscle tissue is frequently observed, which can interfere with insulin signaling pathways and compromise amino acid metabolic processes. Such disruptions may lead to systemic lipid dysregulation, impair muscle blood flow and oxygen consumption, and consequently accelerate the progression of sarcopenia.^12^ Furthermore, excessive adipose deposition can activate chronic inflammatory mechanisms, promoting the secretion of pro-inflammatory cytokines, including tumor necrosis factor, leptin, and growth hormone. This cascade exacerbates muscle catabolism and insulin resistance, forming a self-sustaining cycle that intensifies both fat accumulation and skeletal muscle loss.^29^

To further evaluate the robustness of the association between the CMI and sarcopenia, this study carried out subgroup analyses based on variables such as gender, age, BMI, race, comorbidities including hypertension and diabetes, and PA level. Interaction effects among subgroups were also investigated. The findings revealed a statistically significant relationship between elevated CMI and increased odds of presenting sarcopenia in the overall population (OR = 1.28, 95 % CI: 1.16–1.41, *p* < 0.001). This association remained largely consistent across the majority of subgroups. A statistically significant interaction was identified only for BMI (interaction *p* = 0.009), whereas no meaningful interactions were detected for other covariates.

Specifically, among individuals with a BMI below 25 kg/m^2^, each one-unit increase in CMI was associated with a 2.28-fold elevated odds of presenting sarcopenia (*p* = 0.002). Conversely, no statistically significant association was identified in individuals with a BMI ranging from 25 to 29.9 kg/m^2^, and only a marginal increase in risk was observed for those with a BMI of 30 kg/m^2^ or higher (OR = 1.10, *p* = 0.016). This observed trend may be attributed to the fact that variations in muscle mass within the normal BMI category are more indicative of overall health status. In contrast, in overweight or obese individuals, excessive adipose tissue may obscure true alterations in muscle mass. These findings are consistent with a study conducted in Taiwan, which reported that individuals with lower muscle mass frequently exhibit increased WC.^30^ Likewise, Zhang Tian et al. emphasized that even among individuals with a normal BMI, abdominal obesity may heighten the odds of presenting sarcopenia in older populations.^7^

Sarcopenia is traditionally regarded as an age-related disorder predominantly affecting older adults. However, recent evidence suggests that the reduction in muscle mass may initiate earlier in the lifespan.^16^ A prior investigation focusing on U.S. adults aged ≥ 60-years demonstrated that higher CMI values were inversely associated with sarcopenia prevalence.^31^ In contrast, the present study focused on individuals aged 20 to 59 years and further substantiated the relationship between CMI and sarcopenia within distinct age groups through stratified subgroup analysis.

It is important to highlight that the association between CMI and sarcopenia was significantly stronger in individuals aged 20–39 years compared to those aged 40–59 years. This disparity may be explained by the higher prevalence of metabolic disturbances and obesity within the younger adult population. Lifestyle patterns, dietary habits, and metabolic conditions in this age group may increase their susceptibility to fluctuations in CMI, potentially resulting in insulin resistance and dyslipidemia, which subsequently contribute to increased fat accumulation and skeletal muscle loss. In contrast, among individuals over the age of 40, the effects of chronic diseases, pharmacological treatments, and genetic predispositions on sarcopenia become more prominent, likely reducing the relative influence of CMI. These observations suggest that CMI represents a key risk factor for sarcopenia in younger adults, while in middle-aged and elderly individuals, the cumulative impact of multiple variables plays a more dominant role. Consequently, implementing targeted CMI monitoring and intervention strategies during early adulthood may be essential for preventing premature skeletal muscle decline.^32^

This study further demonstrated that individuals diagnosed with sarcopenia exhibit a higher predisposition to comorbidities such as diabetes and hypertension. Subgroup analysis revealed a more pronounced association between CMI and the odds of presenting sarcopenia specifically within the hypertensive cohort. Among non-diabetic participants, a positive correlation between CMI and sarcopenia was observed; however, this relationship did not reach statistical significance in diabetic patients (*p* > 0.05). These results are consistent with the findings of Zhang Ke et al., who investigated an elderly hypertensive population in Urumqi and documented a greater incidence of sarcopenia in hypertensive individuals compared to non-hypertensive controls. This disparity may be attributable to microvascular sclerosis and compromised muscle oxygen delivery induced by chronic hypertension.^33^ Furthermore, Mattace-Raso et al. proposed that hypertension is associated with a persistent state of low-grade inflammation, wherein inflammatory biomarkers are significantly implicated in the progression of sarcopenia, potentially representing a critical mechanistic link.^34^

Epidemiological investigations in Japan have identified a notably high prevalence of sarcopenia among diabetic patients, with emerging evidence suggesting that glycemic variability constitutes an independent risk factor for the condition.^35^ Research by Kalyani et al. has established glycosylated Hemoglobin (HbA1c), a well-established biomarker of chronic hyperglycemia, as significantly correlated with progressive declines in muscular strength and an essential parameter in sarcopenia evaluation^36^ Ramasamy et al. emphasized that the accumulation of Advanced Glycation end products (AGEs) in diabetic individuals can induce inflammatory responses within skeletal muscle tissue, thereby contributing to reductions in both muscle mass and strength via collagen cross-linking pathways.^37^ Furthermore, metabolic dysregulation and restrictions on PA are frequently observed in diabetic patients, which may impair the processes of muscle formation and maintenance. The absence of a statistically significant association between CMI and odds of presenting sarcopenia within the diabetic subgroup may be attributable to insufficient statistical power due to the limited sample size.

This investigation further demonstrated that individuals diagnosed with sarcopenia generally display reduced levels of PA. Subgroup analysis indicated a positive correlation between CMI and odds of presenting sarcopenia among those participating in vigorous exercise, potentially due to the exacerbation of muscle damage and heightened inflammatory responses associated with elevated CMI levels during high-intensity activity. Conversely, in individuals engaging in moderate-intensity exercise, improved metabolic adaptability may serve as a protective factor, attenuating the negative impact of high CMI and rendering the association non-significant. Among those with minimal PA, the lack of sufficient muscular stimulation and diminished metabolic demand may diminish the strength of the relationship between CMI and odds of presenting sarcopenia, although the overall risk remains elevated. Chronic physical inactivity has been shown to induce disuse-induced muscle atrophy, thereby promoting the onset of sarcopenia.^38^ A population-based observational study in Taiwan corroborated these findings, reporting that inadequate PA could lead to a reduction in quadriceps mass by up to 6.4 %.^39^ Furthermore, it is well documented that a structured regimen combining regular resistance training with aerobic exercise can markedly reduce skeletal muscle apoptosis, improve muscle fiber architecture, decrease the likelihood of falls, and contribute to better regulation of glucose and lipid homeostasis.^40^

This investigation exhibits multiple significant strengths. First, the data were sourced from the widely recognized NHANES database, which utilizes a rigorous multi-stage probability sampling methodology and applies sample weights to ensure the representativeness and broad applicability of the study outcomes. Second, the substantial sample size facilitates detailed subgroup analyses, thereby strengthening the validity and robustness of the conclusions. To the best of our knowledge, this is the first comprehensive study to explore the relationship between the CMI and the prevalence of sarcopenia specifically in the young and middle-aged U.S. population, highlighting CMI’s potential as an effective biomarker for assessing the odds of presenting sarcopenia. The statistical analysis identified a non-linear positive association between CMI and sarcopenia, with a critical threshold value determined at 0.899. Multidimensional prediction models constructed based on the CMI demonstrate superior discriminative performance (AUC = 0.819) compared to conventional anthropometric indices such as BMI and WC. Stratified subgroup analyses further validated the relevance of CMI across distinct population segments, providing innovative perspectives and analytical frameworks for the development of precision medicine strategies.

Despite the multiple merits of the present study, it is imperative to acknowledge its inherent limitations. Firstly, the cross-sectional design restricts causal inference; future longitudinal studies are warranted to verify the temporal association between CMI and sarcopenia. Secondly, the assessment of muscle mass using DXA limits the eligible population to individuals aged 20–59 years, resulting in the exclusion of those aged 60 and above. Furthermore, the technical constraints of DXA regarding subject body size and mobility may systematically preclude severely obese or physically impaired individuals, thereby introducing selection bias. This methodological limitation may compromise the generalizability of our findings to the broader population and potentially lead to an underestimation of the true association between CMI and sarcopenia among individuals with extreme body compositions. Additionally, although the authors adjusted for a range of potential confounding factors in our statistical analyses, the possibility of residual confounding cannot be entirely dismissed. Lastly, given that the data were derived from the NHANES, the applicability of our conclusions to populations with diverse racial and cultural backgrounds requires further validation.

## Conclusions

This study identifies a significant link between CMI and sarcopenia prevalence among U.S. adults aged 20–59, with stronger associations observed in males, individuals aged 20–39, non-Hispanic Blacks, those with hypertension, and non-obese/non-diabetic subjects. Elevated CMI levels correspond to increased odds of presenting sarcopenia, with a threshold at 0.899. After adjusting for multiple confounders, the multidimensional prediction model constructed based on CMI demonstrated superior discriminative ability (AUC = 0.819), with evaluation performance surpassing conventional anthropometric indicators. This suggests that CMI holds potential clinical application value as a biomarker for sarcopenia risk assessment. Despite certain methodological limitations in the present study, the aforementioned findings still provide crucial insights for the precision prevention and management of sarcopenia.

## Ethics statement and consent to participate

The NHANES survey protocol, as approved by the NCHS Research Ethics Review Committee (Protocol #2011–17, Continuation of Protocol #2018–01https://www.cdc.gov/nchs/nhanes/irba98.htm), ensured that all study participants provided informed written consent. Since the NHANES database is publicly accessible, this study was exempt from additional ethical review.

## Clinical trial number

Not applicable.

## Consent for publication

Not Applicable.

## Data availability statement

Publicly available datasets were analyzed in this study. These data can be found here: https://wwwn.cdc.gov/nchs/nhanes/search/default.aspx.

## Authors' contributions

J.L and S.B conceptualized the current study. J.L undertook the collection and analysis of the data. Both J.L and S.B collaborated on drafting the initial manuscript. J.L refined the final draft. All authors concurred with the submission of the article to the chosen journal and are committed to assuming responsibility for all facets of the work.

## Funding

The author(s) declare that no financial support was received for the research, authorship, and/or publication of this article.

## Declaration of competing interest

The authors declare that the research was conducted without any commercial or financial relationships that could be perceived as a potential conflict of interest

## Appendix Supplementary materials

Image, application 1
