# Supplementary material for: Cardiometabolic index as a predictor of sarcopenia in U.S. adults aged 20–59: results from a cross-sectional study
Source: Clinics (Sao Paulo). 2026 Mar 31;81:100912. doi: 10.1016/j.clinsp.2026.100912 (PMC13068590; doi:10.1016/j.clinsp.2026.100912)
Supplement: Supplementary file 1 [file mmc1.pdf]

Supplementary Table 1. Comparison of baseline data for sarcopenia classification using PSM.

	Non-sarcopenia	Sarcopenia	p
gender (%)			1

male	213 (50.12)	213 (50.12)	
female	212 (49.88)	212 (49.88)	
age (mean (SD)) (years)	44.14 (10.99)	44.28 (10.95)	0.8513
race (%)			0.9994
Mexican American	144 (33.88)	141 (33.18)	
Other Hispanic	69 (16.24)	70 (16.47)	
Non-Hispanic White	121 (28.47)	121 (28.47)	
Non-Hispanic Black	22 (5.18)	22 (5.18)	
Other Race	69 (16.24)	71 (16.71)	
education (%)			0.0015
Under high school	50 (11.76)	80 (18.82)	
Completed high school	69 (16.24)	70 (16.47)	
College or above	306(72.00)	275(64.71)	
Marital.status (%)			0.4925
Married	233 (54.82)	231 (54.35)	
Widowed	11 (2.59)	7 (1.65)	
Never married	181(42.59)	46 (44)	
alcohol (%)			NaN
Yes	313 (77.09)	287 (71.75)	
No	93 (22.91)	113 (28.25)	
Hypertension (%)			0.0031
Yes	102 (24.00)	142 (33.41)	
No	323 (76.00)	283 (66.59)	
Diabetes (%)			0.0006
Yes	34 (8.00)	65 (15.29)	
No	384 (90.35)	345 (81.18)	
Unclear	7 (1.65)	15 (3.53)	
smoke (%)			0.4437
Yes	182 (42.82)	170 (40.00)	

No	243 (57.18)	255 (60.00)	
CAD (%)			0.3399
Yes	3 (0.71)	7 (1.65)	
No	422 (99.29)	418 (98.35)	
cancer (%)			0.8648
Yes	17 (4.00)	19 (4.47)	
No	408 (96.00)	406 (95.53)	
asthma (%)			0.0649
Yes	52 (12.24)	72 (16.94)	
No	373 (87.76)	353 (83.06)	
CMI (mean (SD))	0.81 (1.63)	1.15 (1.53)	0.0014
PA (%)			0.9645
Vigorous	59 (13.88)	57 (13.41)	
Moderate	52 (12.24)	54 (12.71)	
Mild	314 (73.88)	314 (73.88)	
PIR (%)			<0.0001
＜1.3	107 (27.94)	165 (42.97)	
≥1.3＜3.5	154 (40.21)	136 (35.42)	
≥3.5	122 (31.85)	83 (21.61)	
Protein (mean (SD)) (g)	5.39 (7.99)	5.60 (10.37)	0.7425
Carbohydrate (mean (SD)) (g)	16.06 (20.37)	18.20 (30.62)	0.2442
Dietary_fiber (mean (SD)) (g)	1.09 (1.87)	1.10 (1.93)	0.9663
fat (mean (SD)) (g)	4.84 (8.00)	4.96 (8.81)	0.8424
Energy (mean (SD)) (kcal)	129.28 (161.52)	138.53 (189.62)	0.4561
sugar (mean (SD)) (g)	6.10 (11.56)	8.37 (23.63)	0.0832
Serum creatinine (mean (SD))(umol/L)	78.37 (49.27)	68.80 (21.53)	0.0905
WC (mean (SD)) (cm)	97.53 (13.64)	107.55 (17.33)	<0.0001
BMI (mean (SD)) (kg/m^2)	28.49 (5.68)	33.74 (7.68)	<0.0001

HDL (mean (SD)) (mmol/L)	1.35 (0.37)	1.26 (0.36)	0.0002
TC (mean (SD)) (mmol/L)	5.06 (1.17)	5.16 (1.06)	0.1815
TG (mean (SD)) (mmol/L)	1.54 (2.44)	1.75 (1.49)	0.1233
SAR (mean (SD))	0.79 (0.19)	0.61 (0.14)	<0.0001

For continuous variables:(N) Mean ± SD, Standardized difference= abs(Mean1-Mean0)/ sqrt((S1+S2)/2)

For categorical variables: N (%), Standardized difference = abs(P1-P0)/sqrt((P1*(1-P1)+P0*

CAD Coronary artery disease, CMI Cardiometabolic Index, PIR Ratio of family income to poverty, PA Physical Activity, TC Total Cholesterol, TG Triglycerides, HDL-C High-Density Lipoprotein Cholesterol, WC Waist Circumference, BMI Body Mass Index, SAR Sarcopenia Index.

Supplementary Table 2. Comparison of baseline data for CMI classification by using IPTW.

Characteristic	lower	higher	p
gender (%)			
Male	42.83	67.01	<0.0001
Female	57.17	32.99	
age (years)	38.26 (12.00)	39.86 (11.72)	0.1419
race (%)			0.019
Mexican American	9.26	12.44	
Other Hispanic	6.72	9.84	
Non-Hispanic White	59.85	62.62	
Non-Hispanic Black	13.30	6.26	
Other Race	10.87	8.84	
education (%)			0.275
Under high school	5.24	6.30	
Completed high school	9.63	12.33	
College or above	85.12	81.37	

Marital.status (%)			0.4498
Married	50.01	49.44	
Widowed	0.72	1.33	
Never married	49.26	49.22	
alcohol (%)			0.5467
Yes	80.89	78.55	
No	19.11	21.45	
Hypertension (%)			<0.0001
Yes	20.13	35.46	
No	79.87	64.54	
Diabetes (%)			<0.0001
Yes	3.59	14.78	
No	94.52	81.04	
Unclear	1.89	4.18	
smoke (%)			0.0212
Yes	37.98	47.53	
No	62.02	52.47	
CAD (%)			0.0124
Yes	0.59	1.78	
No	99.41	98.22	
cancer (%)			0.3555
Yes	3.65	4.93	
No	96.35	95.07	
asthma (%)			0.739
Yes	17.64	18.82	
No	82.36	81.18	
CMI (mean (SD))	0.38 (0.18)	1.56 (1.46)	<0.0001
PA (%)			0.5984
Vigorous	16.70	13.86	

Moderate	12.40	11.73	
Mild	70.91	74.41	
PIR (%)			0.2834
＜1.3	26.60	30.56	
≥1.3＜3.5	36.14	38.75	
≥3.5	37.26	30.69	
Protein (mean (SD))(g)	5.09 (8.18)	5.56 (9.58)	0.4832
Carbohydrate (mean (SD)) (g)	17.19 (23.36)	19.76 (30.93)	0.1523
Dietary_fiber (mean (SD)) (g)	1.07 (1.78)	1.25 (2.07)	0.2095
fat (mean (SD)) (g)	4.72 (8.18)	5.40 (10.63)	0.4675
Energy (mean (SD))(kcal)	132.03 (166.29)	149.29 (203.87)	0.2614
sugar (mean (SD)) (g)	7.56 (15.71)	8.72 (23.91)	0.3479
Serum creatinine (mean (SD))	69.73 (18.80)	76.97 (22.20)	0.0272
WC (mean (SD))(cm)	96.60 (16.67)	113.45 (16.41)	<0.0001
BMI (mean (SD)) (kg/m^2)	29.00 (7.31)	35.17 (7.71)	<0.0001
HDL (mean (SD)) (mmol/L)	1.49 (0.37)	1.05 (0.21)	<0.0001
TC (mean (SD)) (mmol/L)	4.81 (0.93)	5.18 (1.12)	<0.0001
TG (mean (SD)) (mmol/L)	0.91 (0.33)	2.27 (1.65)	<0.0001
SAR (mean (SD))	0.74 (0.21)	0.72 (0.17)	0.081

P-value were from weighted t test for continuous variables, and weighted chi-square test for categorical variables

For continuous variables, Standardized difference = abs(Mean1-Mean0)/sqrt((S1+S2)/2)

For categorical variables, Standardized difference = abs(P1-P0)/sqrt((P1*(1-P1)+P0*(1-P0))/2)

CAD Coronary artery disease, CMI Cardiometabolic Index, PIR Ratio of family income to poverty, PA Physical Activity, TC Total Cholesterol, TG Triglycerides, HDL-C High-Density Lipoprotein Cholesterol, WC Waist Circumference, BMI Body Mass Index, SAR Sarcopenia Index.

Table4 Subgroup regression analysis between CMI index with sarcopenia prevalence.

Variable	Percent	OR	Lower	Upper	P	P for interaction
Overall	100	1.28	1.16	1.41	<0.001	
Stratified by gender						0.966
female	50.4	1.26	0.75	2.12	0.391	
male	49.6	1.27	1.14	1.42	<0.001	
Stratified by age(years)						0.674
20-39	49.9	1.31	1.14	1.5	<0.001	
40-59	50.1	1.24	1.05	1.45	0.012	
Stratified by race						0.667
Mexican American	15	1.2	1.06	1.36	0.009	
Other Hispanic	10.7	1.15	0.88	1.51	0.303	
Non-Hispanic White	35.3	1.28	1.15	1.44	<0.001	
Non-Hispanic Black	20	1.78	1.34	2.37	<0.001	
Other Race	19	1.26	1.09	1.46	0.003	
Stratified by BMI(kg/m^2)						0.009
<25	33.1	2.28	1.38	3.75	0.002	
25-29.9	31.4	1.18	0.99	1.39	0.067	
>=30	35.5	1.1	1.02	1.2	0.016	
Stratified by diabetes						0.625
Yes	7.9	1.05	0.93	1.17	0.447	
No	90.4	1.4	1.19	1.63	<0.001	
Unclear	1.7	0.98	0.79	1.23	0.888	
Stratified by hypertension						0.389
Yes	23.9	1.31	1.12	1.53	0.001	
No	76.1	1.2	1.04	1.37	0.013	

Stratified by PA						0.28
Vigorous	17.5	1.48	1.21	1.61	<0.001	
Moderate	12.4	1.12	0.97	1.33	0.122	
Mild	70	1.28	1.15	1.44	<0.001	

BMI: Body Mass Index , PA Physical Activity.
